# Water quality assessment of Elgo river in Ethiopia using CCME, WQI and IWQI for domestic and agricultural usage

**DOI:** 10.1016/j.heliyon.2023.e23234

**Published:** 2023-12-09

**Authors:** Duop Chan Kujiek, Zenebe Amele Sahile

**Affiliations:** a Department of Civil Engineering, University of Juba, Sudan; b Faculty of Water Supply and Environmental Engineering, Arba Minch University, P.O. Box. 21, Arba Minch, Ethiopia

**Keywords:** CCME, WQI, IWQI, Elgo river, Ethiopia

## Abstract

The increasing demand for water due to the escalation in population and aggressive agricultural activities for drinking and irrigation purposes in the rural areas of Ethiopia has put tremendous stress on water requirements. The Elgo River in southern Ethiopia is deteriorating due to sedimentation, soil erosion, stormwater runoff, and anthropogenic activities. Elgo village faces water shortages and a lack of safe drinking water. The purpose of this research was to identify the extent of pollution in Elgo River water using the Canadian Council of Ministers of the Environment (CCME), Water Quality Index (WQI), and Irrigation Water Quality Index (IWQI). A total of 12 water samples were collected from 3 river sampling sites for the dry and wet seasons to test the physicochemical and biological parameters. Results obtained were: turbidity (46.5–156) NTU, colour (103.65–606.5) TCU, EC (182–268) μS/cm, TDS (192.5–275.5) mg/l, TSS (680–2774) mg/l, Ca2+ (22–45) mg/l, Mg2+ (19.5–23.5) mg/l, Cl- (10.5–16.65) mg/l, and SO42- (17.18–47) mg/l for both the dry and wet seasons, respectively. The CCME WQI revealed that the overall results were 38.38 for the dry season and 36.6 for the wet season for drinking water parameters. The CCME WQI categorization indicates that the Elgo River water is classified as poor, with results ranging from 0 to 44. For irrigation purposes 10, parameters such as SAR, PS, PI, MAR, KI, RSC, EC, SSP, TH, and %Na were examined to compute indices using the IWQI model. The overall result of water quality indicated that IWQIs of 81.4 and 62.14 are good for the dry season and poor for the wet season, respectively. This research provides a thorough analysis through modelling to determine the suitability of water for different purposes for the tribal and backward communities of the area.

## 1 Introduction

Surface water is extensively used to meet requirements across the globe, and the quality of the water is crucial for sustainability [1]. One-third of the world's population consumes surface water for domestic, agricultural, and industrial purposes [2]. Surface water pollution in developing nations, which is brought on by both natural and human activity, poses serious health risks and degrades the quality of drinking water [3].

Anthropogenic activities deteriorate the quality of water, especially for industrial, drinking, and irrigation purposes [4]. Water pollution status is determined by evaluating physical, chemical, and bacteriological parameters. Physico-chemical properties measure water quality's influence on water use [5]. Monitoring parameters depend on usage needs and purity [6]. Common indicators for irrigation include pH, conductivity, sodium, potassium, nutrients, and specific compounds [7]. Odour, colour, taste, and other sensible qualities normally determine the portability and safety of water in terms of health and its acceptability to customers. The quality of water is a basic criterion to judge its usability for any purpose and use since it plays a direct role in human health and the welfare of society. The primary reasons for the water quality deterioration are natural and anthropogenic agents. Sedimentation and erosive factors, water-rock interaction, agricultural and industrial activities, mining, contaminated sewage disposal, fishing, deforestation, and other commercial activities are a few of the natural and human-induced factors that affect water quality [8]. Similarly, Elgo River water quality deterioration is caused by natural and anthropogenic factors, including soil erosion, contaminants, municipal waste, industrial effluent, and agricultural runoff. Previously, studies used only one approach: either drinking water quality alone or irrigation water quality alone [[9], [10], [11], [12]]. However, in this study, we tried to combine CCME and IWQI to assess pollution extent, while irrigation water quality indices provide information on suitability [13,14]. The primary objective of this research was to identify the magnitude of pollution for drinking and irrigation purposes in Elgo River water using CCME, WQI, and IWQI indices.

## 2 Materials and methods

### 2.1 Description of the study area

**Fig. 1:**
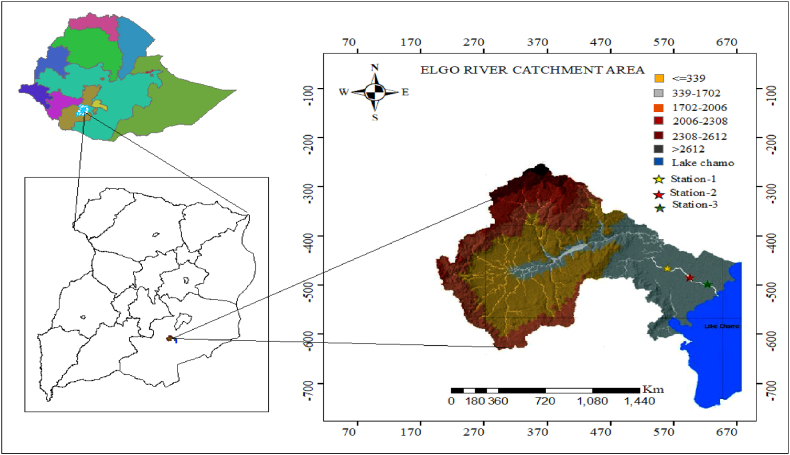
Location map of the study area.

The study area is in the SNNPR Gamo Zone, Arba-Minch, located in the Elgo catchment of the southern Ethiopian Rift Valley. The catchment area is 312 km^2^ and has terrain ranging from 2899 m to 1094 m, with a mean precipitation of 1033 mm. The basin is characterized by a high rate of evaporation, approximately 2300 mm per year on average. The study area is situated between 37°15'23" and 37°30'41" East longitude and 5°45'60" and 5°57'35.5" North latitude. The study area is found in Ethiopia's main rift valley, which features a gently undulating plain surrounded by mountains and ridges and Lake Chamo. Its landscape is characterized by dissected slopes, tectonic processes, and external erosion [15]. The minimum monthly temperature is 14.5 °C, while the maximum monthly temperature is 33.3 °C in the catchment area. The basin experiences bimodal rainfall during the extremely rainy season, and the annual river basin volume is 247,435,316 m^3^. The drainage pattern takes on numerous shapes and behaviours depending on the soil type, lithology, geology, and structures, but dendritic drainage patterns are predominant in the area. The Elgo River basin comprises volcanic rocks and alluvial sediments blanketed with alluvium, constituting the greater part of the area with an elevation less than 1200 m above sea level (Fig. 1).

### 2.2 Sample site selection and sampling

A detailed field visit was commenced along the upstream (S1), middle stream (S2), and downstream (S3) of the river extending up to Chamo Lake before finalizing the exact sampling points. The village is 2 km upstream from a middle stream, which is at the point of contamination under a bridge, and 2 km downstream towards Lake Chamo. Water samples were collected bimonthly in August and September, for the wet season, and November and December, for the dry season, using grab sampling techniques at 30 cm depth manually. Two samples from each point have been collected, which makes six samples for the dry season once in every two weeks period between the two months on the first day of the weeks just for ease of consistent work scheduling, and this has been repeated for the rainy season, so a total of 12 water samples were analyzed. Water samples were collected in 2-L polyethylene bottles, rinsed with HCl, and sealed. High-grade plastic bottles were used for physicochemical and bacteriological analysis and 100-ml glass bottles for biological analysis. Samples were analyzed on-site for pH, EC, TDS, and DO using the HQ400 Multimeter, and for other parameters, samples were transported with an ice box to the Arba-Minch University laboratory and preserved at 4 °C. All samples were analyzed according to the standard procedures of APHA, both for dry and wet seasons.

### 2.3 Water quality analysis

The physico-chemical and biological parameters such as Electrical Conductivity (EC), Turbidity, Power of Hydrogen (pH), Total Dissolved Solids (TDS), Dissolved Oxygen (DO), Biochemical Oxygen Demand (BOD5), Chemical Oxygen Demand (COD), Total Hardness (TH), Calcium (Ca), Magnesium (Mg), Nitrate (NO3-), Nitrite (NO2-), Phosphate (PO43-), Sulphate (SO42-), Chloride (Cl-), Potassium (K+), Colour, Alkalinity, Total Suspended Solid (TSS), Fluoride (F), Iron (Fe), Total Coliform (TC), Faecal Coliform (FC), Sodium (Na+), Carbonate, and Bicarbonate were used for calculating the Water Quality Index [16]. The details of parameters, methods, and reagents used have been tabulated under Table 1.

**Table 1:** Water quality analysis and their respective methods and instruments.

Parameters	Unit	Measurement Site	Instrument/Methodology	Chemicals/Reagent used
pH	….	Onsite	PH Meter	Buffer pH 4, Buffer PH 7, and Buffer 10
**Electrical Conductivity**	μS/cm	Onsite	EC Meter	Standard (500 μs/cm), Standard (1000 μs/cm), and Standard (1500 μs/cm)
**Dissolved Oxygen**	mg/L	Onsite	DO Meter	
**Total Dissolved Solid**	mg/L	Onsite	TDS Meter	
**Colour**	TCU	Laboratory	Colorimetric	Platinum–Cobalt Method
**Chloride**	mg/L	Laboratory	Volumetric analysis	Potassium chromate
**Sulphate**	mg/L	Laboratory	Turbidimetric method	standard sulphate, conditioning reagent, and barium chloride,
**Turbidity**	NTU	Laboratory	Nephelometric	Distilled water
**Alkalinity**	mg/L	Laboratory	Volumetric analysis	H2SO4, methyl orange indicator, and phenolphthalein indicator
**Carbonate alkalinity**	mg/L	Laboratory	Volumetric analysis	
**Bicarbonate alkalinity**	mg/L	Laboratory	Volumetric analysis	
**Total hardness**	mg/L	Laboratory	EDTA TITRIMETRIC METHOD	EDTA, Ammonia buffer, and Eriochrom black indicator
**Calcium**	mg/L	Laboratory	EDTA TITRIMETRIC METHOD	NaOH, Murexide, and EDTA
**Magnesium**	mg/L	Laboratory	EDTA TITRIMETRIC METHOD	EDTA, Eriochrome black, and Ammonia buffer
**Sodium**	mg/L	Laboratory	Flame photo Meter	Standards of sodium, and Distilled water
**Potassium**	mg/L	Laboratory	Flame photo Meter	Standards of potassium, and Distilled water
**Fluoride**	mg/L	Laboratory	DR 28000 spectrophotometer	SPANDS
**Total Iron**	mg/L	Laboratory	UV VIS spectrophotometer	Ammonium thiocyanate, Hydrochloric acid, Potassium permanganate, and Standard ferric solution
**Total coliform**	No/100 ml	Laboratory	Membrane filtration	Distilled water, and Lauryl sulphate Broth
**Fecal coliform**	No/100 ml	Laboratory	Membrane filtration	
**Biochemical Oxygen Demand**	mg/L	Laboratory	Winkler method	Distilled water
**Chemical Oxygen Demand**	mg/L	Laboratory	Open reflex method	H2SO4, Mercuric sulphate, silver sulphate, and K2Cr2O7
**Nitrate**	mg/L	Laboratory	Phenol Di sulphonic acid (Uv VIS spectrophotometer)	Phenol disulfonic acid, Aluminium hydroxide, Standard nitrate solution, and Ammonium hydroxide,
**Phosphate**	mg/L	Laboratory	Ascorbic acid method	Ascorbic acid, Antimony potassium tartrate, and ammonium molybdate
**Nitrite**	mg/L	Laboratory	UV VIS spectrophotometer	Sulphimide,NED
**Total Suspended Solid**	mg/L	Laboratory	Gravimetric method	

### 2.4 Water quality indices (WQI)

WQI incorporates data from multiple water quality parameters into a mathematical equation to express the overall water quality at a certain location and time [11,17]. Each water quality index method has a rating scale to express the status of water quality concerning the index value of the river at selected sampling points [18]. The indexing method has been widely used for better commencement and thorough investigation [19].

#### 2.4.1 Canadian Council members of environment (CCME)

Scope (F1), frequency (F2), and amplitude (F3) are the three major factors that calculate the water quality index using the CCME WQI tool [12] to determine the status of water quality at various sampling points in the water body [20] (Equation (1)). The number of failed tests and failed variables normally influence the water quality index result [9]. Excellent, good, fair, marginal, and poor are the five-scale measures for CCMEWQI (Table 2).

(1) $$\text{WQI}=100\;-(\sqrt{F1^{2}+F2^{2}+F3^{2}}/1.732)$$

Where; F1 = the percentage of water quality indicators not meeting the regulatory guideline values (Scope).

**Table 2:** Irrigation water quality indices and their respective mathematical equations.

Irrigation Water quality Indices	Formula
Sodium Adsorption Ratio (SAR	$\text{SAR}=\frac{{\text{Na}}^{+}}{\sqrt{\frac{{\text{Ca}}^{2+}+{\text{Mg}}^{2+}}{2}}}$
Residual Sodium Carbonate (RSC)	$\text{RSC}=\left({\text{CO}}_{3}^{-2}+{\text{HCO}}_{3}^{-}\right)-\left({\text{Ca}}^{2+}+{\text{Mg}}^{2+}\right)$
Magnesium Adsorption Ratio (MAR)	$\text{MR}=\frac{{\text{Mg}}^{2+}\times 100}{{\text{Ca}}^{2+}+{\text{Mg}}^{2+}}$
Kelly Index (KI)	$\text{KI}=\frac{{\text{Na}}^{+}}{{\text{Mg}}^{2+}+{\text{Ca}}^{2+}}$
Potential Salinity (PS)	$\text{PS}={\text{Cl}}^{-}+\frac{1}{2}\times {\text{SO}}_{4}^{2-}$
Permeability Index (PI)	$\text{PI}=\frac{{\text{Na}}^{+}+{\text{HCO}}_{3}^{-}}{{\text{Ca}}^{2+}+{\text{Mg}}^{2+}+{\text{Na}}^{+}}\times 100$
Sodium Percentage (Na %)	$\text{Na}\;\%=\frac{{\text{Na}}^{+}\times 100}{{\text{Ca}}^{2+}+{\text{Mg}}^{2+}+{\text{Na}}^{+}+K^{+}}$
Total Hardness (TH)	TH = [(2 × $\frac{{\text{Ca}}^{2+}}{40})+\left(2\times \frac{{\text{mg}}^{2+}}{24}\right)]\times 50$
Soluble Sodium Percentage (SSP)	SSP = $\frac{{\text{Na}}^{+}}{{\text{Ca}}^{2+}+{\text{mg}}^{2+}+K^{+}}$ × 100

F2 = the percentage of measurements in which a water quality indicator exceeds the guideline values (Frequency).

F3 = the extent of deviation of the “failed tests” values relative to the corresponding guideline values (Amplitude).

#### 2.4.2 Evaluation of irrigation water quality index

Productivity in agrarian lands and crops usually decreases due to the high concentration of dissolved ions like sodium, carbonate, and bicarbonate in irrigation water, which in turn reduces the quality of agricultural soil both physically and chemically [21]. Those constituents decrease the osmotic pressure in the plant's structural cells by accumulating in the form of ions, preventing water from reaching the branches and leaves. The surface water quality index method for irrigation has its important and is a significant tool in determining the overall impact of the various parameters that are used as a single variable [22]. During the study, various water quality variables (Table 2) were considered to develop the IWQI model by combining the water quality parameters to provide agreeable research [23].

#### 2.4.3 Irrigation water quality indices (IWQI)

IWQI is a dimensionless parameter ranging from 0 to 100, demonstrating the irrigation water quality index for several water quality variables [24]. The irrigation water quality index was calculated by equation (2) and has been compared with five IWQI scales, namely, excellent, good, poor, very poor, and unsuitable (Table 3) ([13,25].

(2) $$IWQI=\sum_{I=1}^{n}W_{cv}*Q_{rv}/\sum_{I=1}^{n}W_{cv}$$

Where;

**Table 3:** Water quality indices and their respective scales.

Canadian Council of Ministers of the Environment Irrigation water quality indices (IWQI)			
WQI value	Rating of the water quality	IWQI Value	Rating of the water quality
95–100	Excellent	85–100	Excellent
80–94	Good	70–85	Good
65–79	Fair	55–70	Poor
45–64	Marginal	40–55	Very Poor
0–44	Poor	0–40	Unsuitable

W_cv_ = relative weight coefficient of the parameters.

Q_rv_ = the recommended standards value of the water quality variable.

## 3 Results and discussion

### 3.1 Physico-chemical water quality results for both seasons (dry and wet)

Twenty-four water quality parameters were analyzed for the collected samples from three sampling stations along the Elgo River (Table 4) based on the standard methods (APHA).

**Table 4:** Seasonal Physico-chemical water quality analysis of Elgo river.

Parameter	S1 Dry S1 Wet		S2 Dry S2 Wet		S3 Dry S3 Wet		WHO	ESA
pH	6.9 ± 0.2	8.4 ± 0.1	7.2 ± 1	8.6 ± 0.1	7.05 ± 0.15	8.5 ± 0.1	6.5–8.5	6.5–8.5
**Turbidity (NTU)**	46.5 ±0.5	36 ± 15	119 ± 15	156 ± 0.5	88 ±1.5	114 ± 0.5	1–5	1–5
**Colour (TCU)**	103.7 ±0.1	436 0	189.5 ± 2.4	606.5 ± 1.0	127 ± 0	382 ± 1.5	10–20	10–15
**EC(μS/cm)**	217 ± 2	182.5 ± 2.5	268 ± 4	187 ± 1.0	246 ± 3	184.5 ± 15	1500	–
**TDS (mg/l)**	226 ± 2	192	275.5 ± 2.5	197 ± 0.1	258 ± 2	193 ± 0.5	500–1500	1000
**Total Solid**	680 ± 1	2642 ± 1	1279 ± 10	2774.5 ±0	1057	2737 ± 7.5	<25	<25
**Total Hardness (mg/l)**	223 ± 2	301 ± 1	231 ± 1	312.5 ± 2.5	226 ± 2	305 ± 1.5	1000	1000
**Total Alkalinity as CaCo**_**3**_**(mg/l)**	107.4 ± 0.6	204.4 ± 2.1	149 ± 6	211.1 ± 1.	126 ± 2	207.1 ± 2.7	200	200
**DO (mg/l)**	6.75 ± 0.1	7.05 ± 0.1	7.35 ± 0.05	7.375 ± 0.025	7. ± 0.1	7.2	5	5
**BOD (mg/l)**	3.55 ± 0.1	2.35 ± 0.1	4.35 ± 0.05	3.35 ± 0.15	3.95 ± 0.05	2.9 ±0.2	120	120
**COD (mg/l)**	36 ± 1.0	28.05 ±0.004	61. ± 12	58±1 1	56 ± 1	45 ± 3	200	200
**Calcium (mg/l)**	22 ± 1.0	23.5 ± 0.1	26.5 ± 1.5	45±3	24.15 ± 085	28 ±1	50	50
**Magnesium (mg/l)**	19.5 ±0.5	23 ± 1	23.5 ±0.5	32 ± 1	21 ±1	29.5 ± 0.5	200	200
**Sodium (mg/l)**	31.5 ±1.5	39 ± 1	35.5 ±0.5	44 ± 1	33 ±1	41.5 ± 1.5	<1.5	<1.5
**Potassium (mg/l)**	2.85 ± 0.1	2.85 ± 0.1	3.5 ± 0.5	3.15 ± 0.05	2.95 ± 0.5	3 ±0.3	<0.3	<0.3
**Iron (mg/l)**	0.1 ± 0.01	0.05 ± 0.1	0.2 ± 0.15	0.235 ±0.005	0.16 ± 0.1	0.16 ± 0.001	250	250
**Chloride (mg/l)**	14.5 ± 0.5	10.5 ± 0.5	17 ± 0.6	12 ± 1.1	15.7 ± 0.7	11.24 ± 0.76	250	250
**Fluoride (mg/l)**	0.5 ± 0.005	0.485 ± 0.005	0.5 ± 0.02	0.675 ± 0.025	0.495 ± 0.015	0.55 ± 0.05	250	250
**Sulphate (mg/l)**	17.18 ± 0.82	35 ± 1	27.43 ± 1.57	47 ± 0.001	19.72 ± 2.28	44.5 ± 0.5	1	1
**Phosphate (mg/l)**	0.1 ± 0.015	0.135 ± 0.005	0.12 ± 0.02	0.2 ± 0.005	0.08 ± 0.01	0.2 ± 0.002	50	50
**Nitrate (mg/l)**	0.2 ± 0.005	0.145 ± 0.005	0.225 = 0.005	0.245 ± 0.005	0.17 ± 0.01	0.2 ± 0.05	50	50
**Nitrite (mg/l)**	0.1 ± 0.01	0.115 ± 0.005	0.195 ± 0.015	0.205 ± 0.015	0.17 ± 0.02	0.17 ± 0.01	3	3
**Total Coliform (CFU/100 ml)**	1207 ± 7	1994.5 ± 5.5	1545.5 ± 3.5	2533.5 ± 33.5	1324.5 ± 3.5	2305.5 ± 38.5	0	0

**Key: S1**: Sampling point at upstream, **S2**: Sampling point at middle stream, **S3**: Sampling point at downstream.

### 3.2 Turbidity

The turbidity value measured for both seasons was calculated to range from 46.5 to 156 NTU (Table 4), which is much above the permissible limit of 5 NTU for drinking purposes (WHO, 2017). The highest average value recorded in the rainy season would be due to increasing the pollution level of the river by washing different vehicles, clothes, and surface runoff at S2 [26]. Due to the disposal of agricultural wastes, domestic wastes, and open excretion, would have contributed to the higher value of turbidity, increasing pollutants posing threats to domestic and irrigation usage.

### 3.3 Colour

The colour values of Elgo River were recorded between 103.65 and 606.5 TCU on average for both seasons, which is above the permissible limit of 20 TC [27]. The probable reason for the higher colour would be the waste from the community to the water sources due to a lack of awareness about proper waste management systems [28]. Results from all sampling points during both sampling events were above the acceptable limit for drinking and had similar higher colour values.

### 3.4 Electrical conductivity (EC)

The EC values of water in the study area range from 182 to 268 S/cm for both seasons (Table 4), which is within the WHO permissible limit of 1000 S/cm. The electrical conductivity of the water samples representing low-saline water for drinking purposes is due to the low concentrations of ions in surface water. As the river temperature increases due to frequent evaporation, it also causes an increase in conductivity unless the river receives enough rain or stream water [28,29].

### 3.5 TDS

The higher TDS value observed in the dry season was due to the high temperatures, which accelerate evaporation and increase ion concentration [30]. The average TDS values of Elgo River (192.5–275.5) mg/l for both seasons are within the permissible limit (Table 4), while the lower concentration observed in the course of the rainy season could be related to the dilution effect caused by rainfall [27,31,32].

### 3.6 Total Suspended Solid (TSS)

The observed values of TSS in both seasons were 680–2774 mg/l, above the permissible value of 25 mg/l for drinking purposes (Table 4). This is due to contamination through decaying bodies in the river, organic particles released into the water, and sediments settled at the lower part of the water source. The higher TSS content normally absorbs energy from the sun, increasing water temperature and subsequently decreasing the level of dissolved oxygen in the water [33].

### 3.7 pH

The pH values of the Elgo River were between 6.85 and 8.6 for both seasons. The value at Station 2 for the wet season was beyond the threshold value (Table 4), which was most likely attributable to the decomposition of organic village garbage [34]. The highest mean value of 8.6 observed at S2 was presumably related to runoff from agricultural lands in addition to waste disposal by the communities [32].

### 3.8 Sulphate (SO_4_^2−^)

The sulphate concentration is due to temporal variation, which was caused by the degradation of weathered soil organic compounds, agricultural runoff, and other human waste [35]. The measured values were 17.18–47 mg/l for both seasons, depicting a range within the permissible limit of WHO guidelines of 250 mg/l. The maximum value during a dry sampling event could be due to the sediments at the bottom of the river that release the ion into the water column or erosional deposits.

### 3.9 Total Hardness (TH)

The TH value, which lies between 223 and 312.5 mg/l measured for both seasons, was within the WHO maximum permissible limit (Table 4). The lowest value of TH measured during the wet and dry seasons was due to the flow rate of the stream and the low amount of both magnesium and calcium ions, respectively [34].

### 3.10 Total alkalinity

The total alkalinity value in Elgo River was in the range of 107.35–211.1 mg/l for both seasons, which is within the WHO permissible limit for the dry season and above the WHO maximum permissible limit for the wet season (Table 4) [36]. Higher concentrations of alkalinity may be due to the salts in water, which, when dissolved, provide higher temperatures in the wet season [37].

### 3.11 Phosphate PO_4_^3-^

A low phosphate concentration was recorded in Elgo (0.07–0.17 mg/l), which is within the WHO maximum permissible limit (Table 4). According to Ref. [19], the lowest value in the rainy season was due to the dilution caused by precipitation, and the minimum value in the dry period could be due to low river flow. As per the [27] standard, the maximum permissible limit of phosphate for drinking is 1 mg/l, and all sample points in the Elgo River were safe for drinking purposes.

### 3.12 Iron (Fe^2+^)

The concentration of iron in the Elgo River ranges from 0.05 to 0.23 mg/l for both seasons (Table 4). During the rainy season, the concentration of iron increased more than during the dry season. The concentration values of iron in the dry season might be caused by the evaporation and reduction of river inflow, while the higher value for the wet season might be due to the untreated domestic wastewater and agricultural runoff water discharge to the river.

### 3.13 Nitrate (NO_3_) and nitrite (NO_2_^−^)

Elgo River has concentrations of nitrate and nitrite of 0.05–0.24 mg/l and 0.13–0.205 mg/l for both seasons, respectively, which were within the lowest limits of WHO values (50 mg/l and 3 mg/l). The lowest values of nitrate and nitrite measured for both seasons would be due to the low anthropogenic influence. The other reason would be the occurrence of less runoff, which carries nitrogen-containing fertilizers from nearby farmland, livestock manure, and domestic waste.

### 3.14 Dissolved oxygen

The dissolved oxygen (DO) concentration in the Elgo River was found in the range of 6.75–7.38 mg/l for both seasons (Table 4), which was below the WHO maximum permissible limit of >7 mg/l at 25 °C. The observed lowest DO concentration was due to the increase of suspended material that affected the dissolution of DO [38]. On the other hand, the presence of organic matter in the water from human activities can result in a reduction in the concentration of DO [39]. Moreover, untreated waste, surface runoff from the area, and fertilizer runoff from agricultural areas along the river may be the reasons for the reduced concentration of DO. More precisely, the DO obtained during the dry season was possibly due to the low water temperature and plant respiration [10].

### 3.15 Biological oxygen demand (BOD)

The BOD values in the Elgo River have been in the range of 3.55–4.35 mg/l, which is within the permissible limit of 5 mg/l (APHA). The lowest value (Table 4) of BOD5 would be related to the low activities of bacteria and fungi to decompose the organic matter, while the higher BOD5 content in the course of the wet season was possibly due to the introduction of wastes in nearby rural areas, including domestic and agricultural wastes. The presence of fertilizers and pesticides used by farmers in their agricultural fields cannot be ruled out.

### 3.16 Chemical Oxygen Demand (COD)

COD values in the Elgo River range from 28.5 to 61 mg/l, which is above the permissible limit of 120 mg/L as per WHO standards (Table 4). COD values were recorded where untreated domestic wastewater entered the river due to urban wastewater and agricultural runoff, which affected human health [40]. The excessive number of organic substances in freshwaters generally originate from domestic sewage, industrial effluents, urban runoff, and farm wastes, which are the primary causes of water pollution [41].

### 3.17 Total coliform, faecal coliform and Escherichia coli (*E. coli*)

The total and faecal coliform contents were higher in the rainy season than in the dry sampled period, respectively. The highest value during a rainy event was probably due to the runoff that carries nitrogen-containing fertilizers from nearby farmland, livestock manure, and domestic waste. Therefore, the bacteriological test results were compared with the value set by Ref. [27] as a drinking water guideline. However, the river showed that the samples taken for both seasons were all above the WHO and Ethiopia's permissible limit for drinking; therefore, there is a significant harmful effect on human health.

### 3.18 CCME Elgo river water quality results for drinking

The 24 parameters analyzed have revealed that 17 out of 24 parameters were within the maximum permissible limit for both seasons except for TSS (680–2774.5 mg/l), colour (103.65–606.5 TCU), turbidity (46.5–156 NTU), K+ (2.85–3.15 mg/l), TC and FC, and alkalinity (204.4–211.1 mg/l). The computed CCME WQI mathematical equation (1) revealed that the overall results were 38.38 for the dry season and 36.6 for the wet season. The river water falls under the poor river water type according to the CCME WQI categorization, as the results ranged from 0 to 44 (Table 3).

### 3.19 Appraisal of river water quality for irrigation

Poor irrigation water quality decreases agricultural crop yields. The mineral composition is signified by the quality of irrigational water, and the quality proves its effects on plants and soil. High-quality and quantity crops yield from the use of high-quality water. The quality of irrigation water is highly variable, depending on both the type and quantity of salts dissolved in it. These salts originate from natural (weathering of rocks and soil) and anthropological (domestic and industrial discharges) sources, and once introduced, they follow the flow path of the water [18,42].

### 3.20 Sodium adsorption ratio (SAR)

The SAR values for the stations range from 6.91 to 7.1 mg/l, with an average value of 6.99 mg/l. On the other hand, the observed values for the rainy season investigation range from 7.87 to 8.1 mg/l, with an average value of 8.0 mg/l. The SAR value of Elgo River was less than 20 in all sample points during both sampling events. As revealed [43]), water with a SAR value greater than 75 and less than 20 is not suitable or favourable for irrigation application, respectively. The Elgo River study result represents an excellent water type for both seasons.

### 3.21 Kelly ratio index (KR)

The KR values for the stations range from 0.71 to 0.76 mg/l, with an average value of 0.73 mg/l. On the other hand, the observed values for the rainy season investigation range from 0.73 to 0.84 mg/l, with an average value of 0.77 mg/l. All sampled water from each point in both study periods was less than one, and, therefore, the water quality of the river from each sampled point for both seasons was appropriate for irrigation purposes. They all fall into suitable river water types, indicating that there is no significant excess of sodium in the surface water.

### 3.22 Residual sodium carbonate (RSC)

The RSC values for the stations range from 59 to 92.5 mg/l, with an average value of 75.12 mg/l. The observed values for the rainy season range from 142.5 to 149.5 mg/l, with an average value of 145.03 mg/l. The RSC value is favourable for irrigation when it is less than 1.25 meq/L (<2.5). A value of RSC between 1.25 and 2.5 meq/L is of marginal quality, whereas a value greater than 2.5 meq/L (>2.5) is not appropriate for irrigation [44]. In the recent study, the water samples were found to have an RSC value greater than 2.5 (>2.5) for both seasons, respectively. The RSC value at all the sampled points for both seasons was greater than 2.5. The water quality from all sampling spots was ‘bad’ for irrigation. They fall into the unacceptable water type category, indicating that there was a marginally excess quantity of sodium in the surface water.

### 3.23 Sodium percentage (% Na)

The Na% values for the stations range from 43.53 to 45.29 %, with an average value of 44.38 %. On the other hand, the observed values for the rainy season investigation range from 44 to 47.37 %, with an average value of 45.29 %. The relatively lowest sodium percentage was calculated for the dry and rainy study periods because of variations in the contents of cations among seasons. However, the sodium percentage from all sampling points for both periods of the study was less than 60 %, and therefore, the river water was suitable for irrigation application [2]. The study revealed that the river water from all sampled spots in the course of both study periods was within the permissible class type.

### 3.24 Magnesium adsorption ratio (MAR)

The MAR values for the stations range from 46.99 to 47 mg/l, with an average value of 46.83 mg/l. Investigations of the observed values for the rainy season range from 49.46 to 53.3 mg/l, with an average value of 51.97 mg/l, respectively. A magnesium ratio greater than 50 % is unsuitable for irrigation use. Conversely, it is suitable if the magnesium ratio is less than 50 %. Based on this, the magnesium ratio of river water from all sample points for the dry season was less than 50 %, whereas the magnesium ratio for the wet season was above 50 %. In general, the sampled water from each point was safe for irrigation during the dry season, as they all fell into an acceptable water type, indicating that there was no significant threat from magnesium in the surface water, while the river water sampled for the wet season fell into an unacceptable water type for irrigation.

### 3.25 Total Hardness (TH)

The TH values for the stations range from 136.25 to 164.42 mg/l, with an average value of 137.89 mg/l for rainy season investigation. TH ranges from 154.58 to 203.3 mg/l, with an average value of 176.96 mg/l. The majority of water samples displayed TH values lying above the standard values used for irrigation suitability. The low values of TH are probably due to the presence of alkaline earth ions (Ca and Mg) of weak acids (HCO3 and CO3) and strong acids (Cl, SO4, and NO3) [23]. Therefore, low alkalinity values reflect the immature hydrochemistry of surface water during seepage and hypodermic flow. The investigation revealed that the river water from all sampled spots in the course of both seasons was in the moderate river water type for the dry season and the hard-class river water type for the wet season.

### 3.26 Soluble sodium percentage (SSP)

The SSP values for the stations range from 66.92 to 71.03 mg/l, with an average value of 68.853 mg/l. On the other hand, the observed values for the rainy season investigation range from 69.68 to 79.03 mg/l, with an average value of 73.216 mg/l. The soluble sodium percentage value of Elgo River was more than 20 mg/l and also more than 60 mg/l in all sample points during both sampled events. Accordingly, the river water was doubtful for irrigation purposes in the course of both seasons, separately. Moreover, the SSP value of the surface water in the study area represents a doubtful water type for irrigation.

### 3.27 Permeability index (PI)

The PI values for the stations range from 54.47 to 60.64 mg/l, with an average value of 57.05 mg/l. The observed values for the rainy season investigation range from 55.21 to 61.15 mg/l, with an average value of 57.63 mg/l, respectively. A permeability index less than 25 mg/l is unsuitable for irrigation, whereas if the PI of water is between 25 and 75 mg/l or above, the water is good for irrigation purposes. However, it will be safer if the permeability index is greater than 75 mg/l. Based on the classification of the permeability index, all the computed values of PI were under the category of excellent for irrigation in both sampled periods at each sample point [8]. Therefore, it can be decided that the water quality of the Elgo River from all sample points was excellent for irrigation use based on the permeability index.

### 3.28 Potential salinity (PS)

The PS values for the stations range from 14.64 to 16.98 mg/l. On the other hand, the observed values for the rainy season investigation range from 20.42 to 23.51 mg/l, with an average value of 22.10 mg/l, respectively. Because of differences in chloride and sulphate concentrations, there was some variance in the computed potential salinity value between sampling points and seasons. The PS value less than 5 mg/l is appropriate for irrigation, and if it is more than 5 mg/l, it is not suitable for crops [43]). The study exposed that the Elgo River water from all sample spots in the course of both study periods was injurious to the unsatisfactory water class type.

### 3.29 Elgo river water quality results for irrigation

The Irrigation Surface Water Quality Index (IWQI) values ranged between 70 and 85 for the dry season and 55 and 70 for the rainy season, with a value of 81.4 and 62.14, respectively, indicating good river water type for the dry season and poor river water type for the wet season, according to the Irrigation Water Quality model [45].

## 4 Conclusions

The research confines itself to calculating the concentrations of most water quality variables during the rainy season compared to the dry term. Mostly at the S2 sample points, there are lots of issues in comparison to the other river sections due to the pollution caused by the community. From the variables, colour, turbidity, TSS, potassium, total coliforms, and faecal coliforms were the indicators that exceeded WHO and Ethiopian guidelines for drinking water in all sampled points for both seasons except alkalinity in the wet season, which was above WHO and ESA maximum permissible limits for drinking and domestic purposes. The computed CCME WQI revealed that the river water quality status for drinking purposes for both seasons was unsuitable as the degree of river water pollution was high. The overall CCME results were 38.38 for the dry season and 36.6 for the wet season, respectively. The river water was unfit for drinking unless appropriately treated. The overall result for irrigation indicated 81.4, which indicates a good river water type for the dry season and 62.14 for a poor river water type for the wet season, respectively. Water quality for irrigation depends on salts and their dissolved quantity. As the total salt concentration increases in various soils, crop development problems result in crop yield reduction and soil erosion. The suitability of water to use for a particular purpose is decided based on its long-term effects and severity. Therefore, the Elgo River water was found to be appropriate for irrigation in both seasons as per irrigation water quality index permission, but a few issues prevailed for drinking purposes. As this study has been done with little data due to financial constraints, we recommend future studies on this area with long-term data measurements and detailed groundwater interactions with pollution sources as well as river researchers. Once the extent of pollution has already been identified in our research, we recommend that further research has be done in the future with integrated methods and techniques that can zoom out point-source pollution to make monitoring of those sources critically is very important, as there is no single magic method to perform all at once.

## Data availability

Data used for this research will be made available upon request. Supplementary data can be found in https://doi.org/10.17632/fszrjndt7s.1.

## Funding statement

The authors are thankful to Arba Minch University for their support in doing this research work.

## CRediT authorship contribution statement

**Duop Chan Kujiek:** Methodology, Investigation, Formal analysis, Data curation, Conceptualization. **Zenebe Amele Sahile:** Writing – review & editing, Writing – original draft, Supervision.

## Declaration of competing interest

The authors declare that they have no known competing financial interests or personal relationships that could have appeared to influence the work reported in this paper.
