# Supplementary material for: Water quality assessment of Elgo river in Ethiopia using CCME, WQI and IWQI for domestic and agricultural usage
Source: Heliyon. 2023 Dec 9;10(1):e23234. doi: 10.1016/j.heliyon.2023.e23234 (PMC10758778; doi:10.1016/j.heliyon.2023.e23234)
Supplement: Multimedia component 1 [file mmc1.pdf]

Supplementary Data

Table S_1: Results of surface water quality analysis for Dry seasons

				S1	S2	S3
S/No	Parameter		Unit	$\bar{x} \pm sd$	$\bar{x} \pm sd$	$\bar{x} \pm sd$
1	potential Hydrogen (P^H)		-	6.85±0.15	7.2±1	7.05±0.15
2	Electrical Conductor		µS/cm	217±2	268±4	246±3
3	Total Dissolve Solid		mg/l	226±2	275.5±2.5	258.5±2.5
4	Color		TCU	103.65±3.35	107.5±2.5	105±3
5	Turbidity		NTU	46.5±0.5	63±2	57.95±105
6	Total Solid		mg/l	680±1	1279±10	1057±8
7	Total Hardness		mg/l	223±2	231±1	226.5±2.5
8	Total Alkalinity		mg/l	107.35±0.65	149.7±2.6	126.05±2.15
	8a	OH	mg/l	6.85±0.15	7.2±0.1	7.05±0.15
	8b	CO_3	mg/l	12±0.1	20. ±1	15.5±0.5
	8c	HCO_3	mg/l	88.5±0.5	122.5±1.5	103.5±1.5
9	Dissolve Oxygen		mg/l	6.75±0.05	7.35±0.05	7. ±0.1
10	Biological Oxygen Demand		mg/l	3.55±0.05	4.35±0.05	3.95±0.05
11	Chemical Oxygen Demand		mg/l	36±1	61. ±12	56±1
12	Calcium		mg/l	22±1	26.5±1.5	24.15±085
13	magnesium		mg/l	19.5±0.5	23.5±0.5	21±1
14	Sodium		Mg/l	31.5±1.5	35.5±0.5	33±1
15	Potassium		mg/l	2.85±0.05	3.05±0.05	2.95±0.05
16	Iron		mg/l	0.045±0.005	0.175±0.015	0.155±0.005
17	Chloride		mg/l	14.5±0.5	16.65±0.65	15.7±0.7
18	Fluoride		mg	0.485±0.005	0.5±0.02	0.495±0.015
19	Sulfate		mg/l	17.18±0.82	27.43±1.57	19.72±2.28
20	Phosphate		mg/l	0.065±0.015	0.12±0.02	0.08±0.01
21	Nitrate		Mg/l	0.155±0.005	0.225=0.005	0.17±0.01
22	Nitrite		mg/l	0.13±0.01	0.195±0.015	0.17±0.02
23	Total Coliform		CFU/100ml	1207±7	1545.5±3.5	1324.5±3.5
24	Fecal Coliform		CFU/100ml	216.5±4.5	261±2	234±3

Table S_2: Results of surface water quality analysis for Rainy season

				S1	S2	S3
**S/No**	**Parameter**		**Unit**	$\bar{x} \pm sd$	$\bar{x} \pm sd$	$\bar{x} \pm sd$
**1**	potential Hydrogen (P^H)		-	8.4±0.1	8.6±0.1	8.5±0.1
**2**	Electrical Conductor		µS/cm	182.5±2.5	187±1	184.5±1.5
**3**	Total Dissolve Solid		mg/l	192±.01	197±0.001	193.5±0.5
**4**	Color		TCU	453.5±2.5	606.5=4.5	563.5±3.5
**5**	Turbidity		NTU	120.5±0.5	156±4	144±3
**6**	Total Solid		mg/l	2642±1	2774.5±2.5	2737.5±7.5
**7**	Total Hardness		mg/l	301±1	312.5±2.5	305.5±1.5
**8**	Total Alkalinity		mg/l	204.4±2.1	211.1±1.1	207.1±2.7
	8a	OH	mg/l	8.4±0.1	8.6±0.1	8.5±0.1
	8b	CO_3	mg/l	19.5±0.5	22.5 ±0.001	20.6±0.6
	8c	HCO_3	mg/l	176.5±1.5	180±1	178±2
**9**	Dissolve Oxygen		mg/l	7.05±0.05	7.375±0.025	7.2±0
**10**	Biological Oxygen Demand		mg/l	2.35±0.05	3.35±0.15	2.9±.020
**11**	Chemical Oxygen Demand		mg/l	28.05±0.004	58 ± 11	45±3
**12**	Calcium		mg/l	23.5±0.05	45 ± 3	28±1
**13**	magnesium		mg/l	23±1	32±1	29.5±0.5
**14**	Sodium		Mg/l	39±1	44±1	41.5±1.5
**15**	Potassium		mg/l	2.85±0.05	3.15±0.05	3±.0030
**16**	Iron		mg/l	0.05±0.001	0.235±0.005	0.16±0.001
**17**	Chloride		mg/l	10.455±0.505	11.995±1.005	11.24±0.76
**18**	Fluoride		mg	0.485±0.005	0.675±0.025	0.55±0.05
**19**	Sulfate		mg/l	35±1	47±0.001	44.5±0.5
**20**	Phosphate		mg/l	0.135±0.005	0.165±0.005	0.15±0.002
**21**	Nitrate		Mg/l	0.145±0.005	0.245±0.005	0.185±0.05
**22**	Nitrite		mg/l	0.115±0.005	0.205±0.015	0.17±0.01
**23**	Total Coliform		CFU/100ml	1994.5±5.5	2533.5±33.5	2305.5±38.5
**24**	Fecal Coliform		CFU/100ml	347±3	580.5±1.5	449±17.5

Table S_3: Relative weight of hydro chemical parameters in the study area for dry season.

S/no	Parameters	Wcv	Qrv	Wcv× Qrv
1	Sodium absorption ratio (SAR)	0.13	77.2	10.036
2	Electrical conductivity (EC)	0.001	0.235	0.00023
3	Kelly's ratio (KR)	1.1	54.4	59.84
4	Residual Sodium carbonate RSC	0.005	3,784.15	18.92
5	Sodium percentage Na%	0.07	182.1	12.75
6	Magnesium absorption ratio (MAR)	0.02	82.35	1.65
7	Total Hardness (TH)	0.013	302.027	3.926
8	Soluble sodium percentage (SSP)	0.05	247.4	12.37
9	Permeability Index (PI)	0.013	0.56	0.0073
10	Salinity potential (SP)	0.2	54.44	10.89
	TOTAL	1.602		130.4
IWQI		81.4		

Table S_4 Relative weight of hydro chemical parameters in the study area for wet season.

S/no	Parameters	Wcv	Qrv	Wcv× Qrv
1	Sodium absorption ratio (SAR)	0.13	63.47	8.25
2	Electrical conductivity (EC)	0.001	198.57	0.2
3	Kelly's ratio (KR)	1.1	4	4.4
4	Residual Sodium carbonate RSC	0.005	105.11	0.53
5	Sodium percentage Na%	0.07	204	14.28
6	Magnesium absorption ratio (MAR)	0.02	71.14	1.42
7	Total Hardness (TH)	0.013	283.9	3.69
8	Soluble sodium percentage (SSP)	0.05	200	10
9	Permeability Index (PI)	0.013	60	0.78
10	Salinity potential (SP)	0.2	280	56
	TOTAL	1.602		99.55
IWQI		62.14		
